# Brain Responses to Violet, Blue, and Green Monochromatic Light Exposures in Humans: Prominent Role of Blue Light and the Brainstem

**DOI:** 10.1371/journal.pone.0001247

**Published:** 2007-11-28

**Authors:** Gilles Vandewalle, Christina Schmidt, Geneviève Albouy, Virginie Sterpenich, Annabelle Darsaud, Géraldine Rauchs, Pierre-Yves Berken, Evelyne Balteau, Christian Degueldre, André Luxen, Pierre Maquet, Derk-Jan Dijk

**Affiliations:** 1 Cyclotron Research Centre, University of Liège, Liège, Belgium; 2 Hololab, Department of Physics, University of Liège, Liège, Belgium; 3 Department of Neurology, Centre Hospitalier Universitaire de Liège (CHU), Liège, Belgium; 4 Surrey Sleep Research Centre, University of Surrey, Guildford, United Kingdom; University of Minnesota, United States of America

## Abstract

**Background:**

Relatively long duration retinal light exposure elicits nonvisual responses in humans, including modulation of alertness and cognition. These responses are thought to be mediated in part by melanopsin-expressing retinal ganglion cells which are more sensitive to blue light than violet or green light. The contribution of the melanopsin system and the brain mechanisms involved in the establishment of such responses to light remain to be established.

**Methodology/Principal Findings:**

We exposed 15 participants to short duration (50 s) monochromatic violet (430 nm), blue (473 nm), and green (527 nm) light exposures of equal photon flux (10^13^ph/cm^2^/s) while they were performing a working memory task in fMRI. At light onset, blue light, as compared to green light, increased activity in the left hippocampus, left thalamus, and right amygdala. During the task, blue light, as compared to violet light, increased activity in the left middle frontal gyrus, left thalamus and a bilateral area of the brainstem consistent with activation of the locus coeruleus.

**Conclusion/Significance:**

These results support a prominent contribution of melanopsin-expressing retinal ganglion cells to brain responses to light within the very first seconds of an exposure. The results also demonstrate the implication of the brainstem in mediating these responses in humans and speak for a broad involvement of light in the regulation of brain function.

## Introduction

Light processing has been studied extensively in the context of circadian biology which emphasizes nonvisual (or non-image-forming) effects of environmental light (irradiance). These nonvisual effects include the synchronization of the circadian system, suppression of melatonin, regulation of sleep, as well as improvements of alertness and cognition [1]–[6]. We have shown that nonvisual responses related to alertness and cognition are associated with changes in regional brain activity detected by positron emission tomography (PET) and functional magnetic resonance imaging (fMRI) [7]–[9]. A number of recent studies, using a wide variety of methodologies, revealed that acute or longer term human nonvisual responses are most sensitive to monochromatic light of wavelengths between ∼460 and 480 nm [2]–[6], [9]–[14]. This sensitivity is much shorter than the overall maximum sensitivity of the photopic system (∼555 nm), and does not coincide with the maximum sensitivity of any of the individual classical photoreceptors (rods: ∼505 nm; S-cones: ∼430 nm; M-cones: ∼530 nm; L-cones: 560 nm) [15], [16].

A fifth retinal photopigment, melanopsin, was recently discovered [17] and shown to be expressed in retinal ganglion cells (RGC) that are intrinsically light sensitive [18], with a maximum sensitivity between 420 to 480 nm [19]–[21]. Melanopsin-expressing RGC are implicated in nonvisual responses to light [18], [22]. They project to numerous brain structures in rodents [23], [24], including hypothalamic nuclei such as the suprachiasmatic nucleus (SCN) and the ventrolateral preoptic area (VLPO), as well as many non-hypothalamic structures including the olivary pretectal nucleus (OPN), and amygdala. Melanopsin-expressing RGC also project to areas typically involved in vision such as the lateral geniculate nucleus (LGN) and the superior colliculus. In addition, melanopsin-expressing RGC project to the LGN and OPN in Macaques [25]. These neuroanatomical pathways provide a mechanism by which irradiance changes could affect many brain functions, *i.e.* circadian entrainment, pupillary constriction, arousal, attention, and emotion regulation, as well as vision [2]–[4], [8], [10], [13], [25], [26]. However, classical visual photoreceptors are necessary to induce complete nonvisual responses to light [27]. In addition, RGC which do not express melanopsin, and presumably are not intrinsically photosensitive, project to the SCN, intergeniculate nuclei (IGL) of the thalamus, and VLPO, suggesting that signal arising from the classical retinal photoreceptors reaches these structures [24], [28]. The relative contribution of the different retinal photoreceptors has not been fully assessed.

Rod and cone responses to light are typically time-locked to the exposure, *i.e.* responses start and cease within a few ms after light is turned on and off, respectively. In addition, quick attenuation of rod and cone signals occurs in the presence of a constant light stimulus [25]. Intrinsic light responses of the melanopsin-expressing RGC are much more sluggish and do not show attenuation: they are only detected seconds after light onset, and firing is maintained for minutes after the end of the light exposure. This feature suggests that these cells are able to account for the long integration time of the nonvisual system [18], [25]. However, melanopsin-expressing RGC receive extrinsic inputs from rods and the three classes of cones, which enable melanopsin-expressing RGC to instantaneously respond to light exposure, and suggest an important role for rods and cones in the nonvisual response to light early in the exposure [25]. Accordingly, assessment of the relative efficacy of different wavelengths indicates that M-cones contribute importantly to the initiation of the response in rodents, but later the melanopsin-expressing RGC are the dominant contributor [13]. Similarly, at lower irradiance classical photoreceptors are sufficient to drive pupillary constriction in rodents while, at higher irradiance, melanopsin-expressing RGC are required to induce a full response [29]. In addition, the wavelength sensitivity of rat SCN neuronal responses to light flashes suggests a contribution of rods and all cones to brief light exposures [30].

A role for S-cones in nonvisual responses in humans was inferred from data showing a greater increase in subjective alertness under violet light exposure (420–440 nm) [31]. However, most human studies investigating the mechanisms of nonvisual responses to light employed monochromatic exposures stimulating most melanopsin-expressing RGC or M- and L-cones, but not S- cones [2], [4], [5], [9]. S-cone contribution to nonvisual responses to light using violet light preferentially triggering these photoreceptors remains to be firmly established. In addition, nonvisual responses to different wavelengths in humans have only been characterized after relatively long duration exposures (at least tens of minutes), *i.e.* presumably after substantial attenuation of rod and cone signals. Thus, the relative contributions of blue, violet and green lights, and by inference of melanopsin-expressing RGC, S- and M-cones, in the establishment of nonvisual responses to light have not been assessed in humans.

Besides the known projections of melanopsin-expressing and non-melanopsin-expressing RGC to brain structures involved in nonvisual functions, most of the brain mechanisms and pathways mediating nonvisual responses to light exposure are unknown. In rodents, the SCN and thalamic IGL receive light irradiance information almost directly and appear therefore to be strongly implicated in eliciting nonvisual responses to light [32], [33]. The SCN and IGL project to many brain structures involved in arousal regulation [33], [34] and a functional indirect connection between the SCN to the brainstem locus coeruleus (LC) has been established [35]. This SCN -brainstem projection may be the pathway by which light modulates alertness. However, beyond these candidate subcortical and brainstem structures, the brain mechanisms involved in generating physiological or behavior nonvisual responses to light have not been characterized in animals.

In humans, using PET and fMRI, we have identified neural correlates of the alerting effects of a bright white light exposure (>7000lux), delivered at night or during the day in brain areas such as the intraparietal sulcus (IPS), hippocampus, thalamic pulvinar, insula, and hypothalamus [7], [8]. More recently we demonstrated that brain activity related to a working memory task is maintained (or even increased) by blue (470 nm) monochromatic light exposure, whereas it decreases under green (550 nm) monochromatic light exposure [9]. These effects were detected in areas implicated in working memory such as the thalamus, insula, IPS, supramarginal gyrus, and middle frontal gyrus (MFG). However, these studies were carried out using relatively prolonged light exposures (17 to 21 min). The brain areas first affected by light exposure, and by inference involved in establishing nonvisual responses to light, are therefore largely unknown in humans.

In the present study we used fMRI to specifically assess early effects of light over the entire brain while participants were performing an auditory working memory task. We used alternating violet (430 nm), blue (473 nm), or green (527 nm) monochromatic light exposures of equal photon density to investigate the processing of stimuli preferentially triggering S-cones, melanopsin-expressing RGC, or M-cones, respectively. Light exposures lasted 50 s, a very short duration from a human circadian biology perspective. We hypothesized that such short duration exposures would induce sustained modulation of the brain responses related to the blocks of the task performed, and that these modulations were wavelength-dependent. This would allow insight in the relative contributions of the different retinal photoreceptors early on in the establishment of nonvisual responses to light. On such a short time scale it is difficult to establish whether the detected brain activity modulations constitute nonvisual or visual responses. This, however, is not essential for our aim, which was to identify brain mechanisms involved in establishing responses to light exposures which eventually will lead to nonvisual responses such as changes in cognition and alertness. We also hypothesized that such short exposures would not induce wavelength-specific responses in a large number of brain areas but would mainly affect a few areas involved in the establishment of the responses, presumably subcortical and brainstem areas. The results support our hypotheses and suggest a prominent role of melanopsin-expressing RGC in the establishment of brain responses to light.

## Methods

### Subjects

Participants were healthy, young subjects (N = 15; 8 females; age: 19–27 [median: 22]; BMI: 18.7–27.3 [median: 22.2]). A semi-structured interview established the absence of medical, traumatic, psychiatric, or sleep disorders. Absence of color blindness was assessed by the 38 plate edition of Ishihara's Test for Color-Blindness (Kanehara Shupman Co., Tokyo, Japan). All participants were non-smokers, moderate caffeine and alcohol consumers, and were not on medication. None had worked on night shifts during the last year or traveled through more than one time zone during the last 2 months. Extreme morning and evening types, as assessed by the Horne-Ostberg Questionnaire [36], were not included. None complained of excessive daytime sleepiness as assessed by the Epworth Sleepiness Scale [37], or of sleep disturbances as determined by the Pittsburgh Sleep Quality Index Questionnaire [38]. All participants had normal scores on the 21 item Beck Anxiety Inventory [39] and the 21 item Beck Depression Inventory II [40]. They were right-handed as indicated by the Edinburgh Inventory [41]. Participants gave their written informed consent and received a financial compensation for their participation. The study was approved by the Ethics Committee of the Faculty of Medicine of the University of Liège.

Volunteers followed a regular sleep schedule during the 7-day period preceding the laboratory segment of the experiment. Compliance to the schedule was assessed using wrist actigraphy (Actiwatch, Cambridge Neuroscience, UK) and sleep diaries. In order to record 2 volunteers on the same day at approximately the same circadian time, volunteers were requested to follow one of 2 sleep schedules differing by 1.5h (2300 h–0700 h +/− 30 min, or 0030 h – 0830 h +/− 30 min). Volunteers were requested to refrain from all caffeine and alcohol-containing beverages and intense physical activity for 3 days before participating in the study.

### Protocol

**Figure 1:**
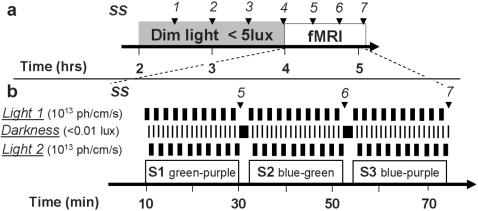
Experimental design. *a.* General timeline. Time relative to scheduled wake time (hrs). *Arrows*: subjective sleepiness assessment (*SS 1-7)*. *b.* Timeline of the fMRI period and light condition organization. Black bars indicate occurrence of the different conditions. Note that the combination of light 1 and 2 changes from one session to the other. *S1-3*: sessions 1 to 3 during which 3 combinations of light are employed (combination order is given as example). Time in minutes after entering the scanner. *Arrows*: subjective sleepiness assessment (*SS 5-7)*.

Subjects were first maintained in dim light (<5 lux) for 2h and then scanned during three consecutive 20 min sessions (Figure 1a). Three drops of tropicamidum 0.5% (Tropicol®) were administered in the eyes 20 min before entering the scanner to inhibit pupillary constriction. In each session, subjects were alternatively exposed to monochromatic 50s light exposures separated by 5-to-14s periods of darkness (<0.01 lux) (Figure 1b). Monochromatic light was violet (430 nm), blue (473 nm), or green (527 nm) and aimed primarily at S-cones, melanopsin-expressing RGC, and M-cones respectively. In each session two wavelengths were presented and alternated. Each color was presented ten times per session.

Subjects were exposed to the three possible combinations of wavelengths over the three sessions. The order of the combinations and the wavelength of the first light exposure in each combination, were counter-balanced over subjects (Supplemental Table S1). In accordance with other protocols in this research area, the photon densities of all light exposures were identical to allow the assessment of the relative contribution of the photoreceptors most sensitive to each wavelength. Photon density was set at 10^13^ photons/cm^2^/s because, at this level, nonvisual responses at night and during the day, depend on the wavelength of the light exposure [2]–[6], [9], [11], [12], [42]. This photon density was equivalent to an illumination level of 4, 7.5, and 24.5 photopic lux for violet, blue and green light exposure, respectively. The first light exposure occurred approximately 4h after habitual wake up time, i.e. during the biological day when melatonin secretion is low [1]. During each session, participants performed an auditory *2-back* working memory task [43], which is reliably executed by a majority of subjects and does not explicitly depend on visual input. Subjective alertness scores, as assessed by the Karolinska Sleepiness Scale (KSS) [44], were collected every 30 min during the 2h preparatory period and between each session while in the scanner.

During the data acquisition period, all subjects interacted with the same investigator who used a standardized set of sentences between each session. This protocol was implemented in order to minimize variation in motivational state due to social interactions [*e.g.* encouragement by an investigator which may modify brain responses [45]]. No feedback was given on performance. Volunteers were trained on a shortened version of the protocol and habituated to the experimental conditions at least a week before the experiment. Subjects had to reach 75% of correct responses on the 2-back task at the end of training to participate to the experiment.

### 2-back-task

Stimuli consisted of nine French monosyllabic consonants that were phonologically different so that they could easily be identified. Stimuli were 500 ms long and the inter-stimulus-interval was 2500 ms. For each consonant, volunteers were requested to state whether or not it was identical to the consonant presented 2 stimuli earlier, by pressing a button on a MR compatible keypad for “yes”, and another one for “no”. Series of stimuli were constructed with ∼30% positive answers. Fourteen consonants were presented in each illumination period for a total of 35s, and 2 to 5 consonants were presented in half of the darkness periods, for a total of 5 to 12.5s. Series could therefore be 33 consonant long if a darkness period with the task was placed between 2 consecutive illumination periods where the task was performed. Series were presented only once and were randomly assigned to one of the scanning sessions. Rest periods could last up to 44 s if a rest period in darkness was placed between two consecutive illumination rest periods. Stimuli were produced using COGENT 2000 (http://www.vislab.ucl.ac.uk/Cogent/) implemented in MATLAB (Mathworks Inc., MA) on a 2.8 GHz XEON DELL personal computer (Round Rock, TX) and were transmitted to the subjects using MR CONTROL amplifier and headphones (MR Confon, Germany). The first session was preceded by a short session during which volunteers had to set the volume level to ensure an optimal auditory perception during scanning.

### Light exposure

Narrow interference band-pass filters (Full Width at Half Maximum [FWHM]: 10nm; Edmund Optic, UK) were used to produce the three monochromatic illuminations. A filter wheel (AB301-T, Spectral Products, NM) was computer controlled to switch band-pass filters and thereby change light wavelength. The light was transmitted by a metal-free optic fiber from a source (PL900, Dolan-Jenner Industries, MA) to two small diffusers placed in front of the subjects' eyes. The diffusers were designed for the purpose of this study and ensured a uniform illumination over the entire visual field. Light was administered through a 4×5.5 cm frame placed 3 cm away from the eye. Spectra of each monochromatic light were checked at the level of the diffusers (AvaSpec-2048, Avantes, The Netherlands), and the 430 nm, 480 nm and 532 nm band-pass filters used produced light with a maximum radiance at respectively 430.3 nm, 472.8 nm and 527.3 nm. Irradiance could not be measured directly in the magnet, but the light source was calibrated and irradiance estimated to be 10^13^ photons/cm^2^/s (840-C power meter, Newport, Irvine, CA) after prereceptoral lens absorption for the different wavelengths was taken into account [46]. The total amount of blue light received during the experiment was well below the blue-light hazard threshold [47].

In order to un-correlate task and light onsets, the auditory task was performed during 35 s of the 50 s illumination periods. Half of the illuminations started with 15 s of rest, the other half terminated with 15 s rest periods. In addition, a 0-to-1 s jitter was implemented between light onset/offset and task onset/offset when they occurred simultaneously in order to further un-correlate them. Darkness periods (<0.01 lux) separated all 50 s illuminations. The auditory task was performed during half of the darkness periods, the duration of which were then 5 to 12.5 s. Rest was requested during the other half; in which case darkness was lasting 9 to 14 s. Illuminations with one color were always followed by darkness periods and then by illuminations in the other color of the session.

### Behavioral data analysis

Accuracy scores were always very high, so we computed d-prime and criterion values following the signal detection theory [48] in order to identify possible changes in behavior not reflected in overall accuracy. Repeated measure ANOVA with light condition and session as within subject factors were carried out separately on d-prime, criterion and reaction times. Repeated measures ANOVA with repetition as within subject factor were computed on subjective sleepiness scores. All behavioral analyses were computed with Statistica 6.1 (StatSoft France, France).

### Functional MRI data acquisition

Functional MRI time series were acquired using a 3T MR scanner (Allegra, Siemens, Germany). Multislice T2*-weighted fMRI images were obtained with a gradient echo-planar sequence using axial slice orientation (32 slices; voxel size: 3.4×3.4×3 mm^3^; matrix size 64×64×32; repetition time = 2130 ms; echo time = 40 ms; flip angle = 90°). The four initial scans were discarded to allow for magnetic saturation effects. There was little variation in the number of scans per session (blue-green sessions: 563.3±5.9 (mean±SD); violet-blue sessions: 563.4±6.2; green-violet sessions: 563.3±7.5). Head movements were minimized using a vacuum cushion. A structural T1-weigthed 3D MP-RAGE sequence (TR 1960 ms, TE 4.43 ms, TI 1100 ms, FOV 230×173 cm^2^, matrix size 256×256×176, voxel size: 0.9×0.9×0.9 mm) was also acquired in all subjects.

### Functional MRI data analysis

Functional volumes were analyzed using Statistical Parametric Mapping 5 (SPM5-http://www.fil.ion.ucl.ac.uk/spm) implemented in MATLAB. They were corrected for head motion, spatially normalized (standard SPM5 parameters) to an echo planar imaging template conforming to the Montréal Neurological Institute (MNI) space, and spatially smoothed with a Gaussian Kernel of 8 mm FWHM. The analysis of fMRI data, based on a mixed effects model, was conducted in two serial steps, accounting respectively for fixed and random effects. For each subject, changes in brain regional responses were estimated using a general linear model in which the different parts of the experimental design were modeled using either boxcar or stick functions, convolved with a canonical haemodynamic response function. Boxcar functions modeled the 15 s rest illumination periods, the 35 s illumination periods including the 2-back task, and the darkness periods during which the task was performed. Stick functions modeled light onsets and light offsets. Melanopsin-expressing RGC do not cease firing at light offset [25], so brain responses to light offsets are unlikely to represent a nonvisual response to light. Further, rest periods during the illuminations were short as compared to the task periods and were contaminated by the performance of the task. The regressors modeling offsets and rest periods were therefore considered as covariates of no interest together with movement parameters derived from realignment of the functional volumes. High-pass filtering was implemented in the matrix design using a cut-off period of 256 seconds to remove low frequency drifts from the time series. Serial correlations in the fMRI signal were estimated using an autoregressive (order 1) plus white noise model and a restricted maximum likelihood algorithm. The effects of interest were then tested by linear contrasts, generating statistical parametric maps. The summary statistic images resulting from these different contrasts were then further smoothed (6mm FWHM Gaussian Kernel) and entered in a second-level analysis. This second step accounts for inter-subject variance in the main effects of light condition (random effects model) and corresponds to a one-sample *t*-test for brain responses to the 2-back series and light onsets. The resulting set of voxel values for each contrast constituted maps of the t statistics thresholded at *p_uncorrected_* = 0.001. Statistical inferences were performed after correction for multiple comparisons on small spherical volumes (svc; 10 mm radius) at a threshold of p_svc_ = 0.05, around *a priori* locations of activation. Activations were expected in structures involved the *n-back* tasks, arousal regulation, and showing nonvisual responses to light in our own fMRI and PET work. Brain areas to which the melanopsin-expressing RGC project or functionally linked to the SCN, were also considered as *a priori* locations of activation. Standard stereotactic coordinates of previously published *a priori* locations, used for svc, are as follow: amygdala: 22 −6 −15 [49]; hippocampus: −30 −30 −2 [8]; LGN: −23 −21 −3 [50]; LC: 2 −32 −20 [51]; thalamus: −14 −14 −16 [9].

## Results

### Behavior

All sessions and light conditions were identical from a behavioral point of view. Statistical analyses showed that performance (reaction times and accuracy) was always high and was not affected by the light condition or sessions (Supplemental Data S1; Figure S1a–c). Computation of subjective sleepiness scores revealed that entering the scanner and the associated change in posture, significantly increased sleepiness. However, bias associated with variations in sleepiness was prevented by the pseudo-randomization of session types (Supplemental Data S1; Figure S1d–f).

### FMRI data

#### Sustained effects

**Figure 2:**
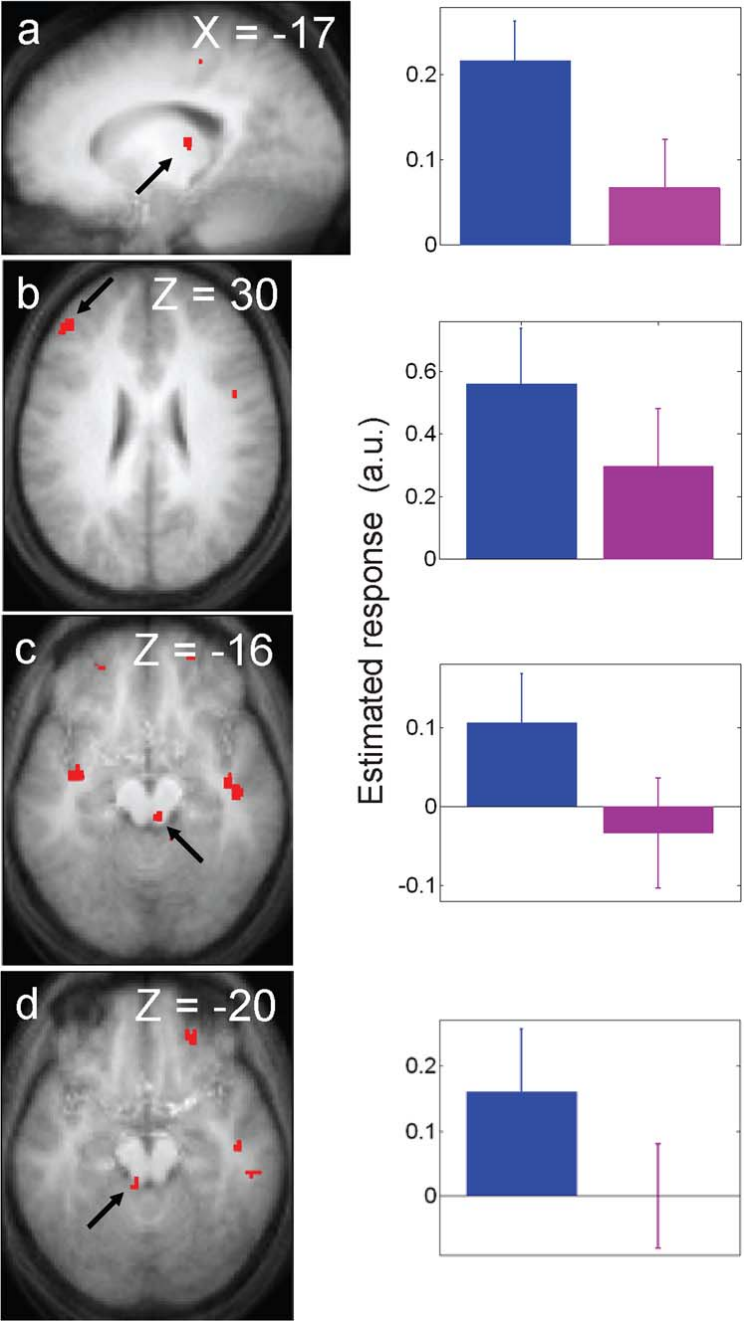
Significant differences between the blue and violet light conditions during the performance of the 2-back task. *Left panels:* statistical results overlaid to the population mean structural image (p*_uncorrected_*<0.001). *Right panels:* Mean parameter estimates of the blue and violet light conditions during the 2-back task (arbitrary units±SEM). *a.* left thalamus–*b.* left MFG–*c.* right brainstem–*d.* left brainstem.

The analysis of fMRI data first focused on the brain responses recorded during the blocks of the 2-back task. The effects described below are therefore sustained because they describe differences between light conditions that were maintained for the duration of the blocks. Significant differences between violet and blue light exposures were detected in the left MFG and in the left thalamus, a few mm away from the location for which we previously found a wavelength dependent effect of light [9], as well as in two areas of the brainstem. Spatial resolution of the fMRI technique does not allow a precise identification of the brainstem nuclei included in the activated areas, but the location of the activations is compatible with several pontine nuclei involved in arousal regulation, and in particular with the LC bilaterally (Figure 2; Table 1) [52]. Activity estimates show (Figure 2; right panels) that, compared to the violet light condition, responses were greater under the blue exposure in these four brain areas. No significant differences between blue and green light exposures, and between violet and green light exposures were detected during task periods (Supplemental Tables S2). Additional analyses suggested that the differences between light conditions were stable during the 50s illuminations (Supplemental Method S1; Supplemental Tables S3).

**Table 1:** Light condition effects during the performance of the task.

*Brain areas*	*xyz*	*Z*	*p*
***Blue light>violet light***			
**Left middle frontal gyrus**	−44 42 30	3.45	0.020
**Left thalamus**	−18 −24 10	3.32	0.028
**Left brainstem**	−6 −38 −20	3.22	0.035
**Right brainstem**	6 −30 −16	3.17	0.040

Coordinates (xyz) in the standard MNI space. No other significant light condition effects were found during the performance of the task.

#### Transient effects

**Figure 3:**
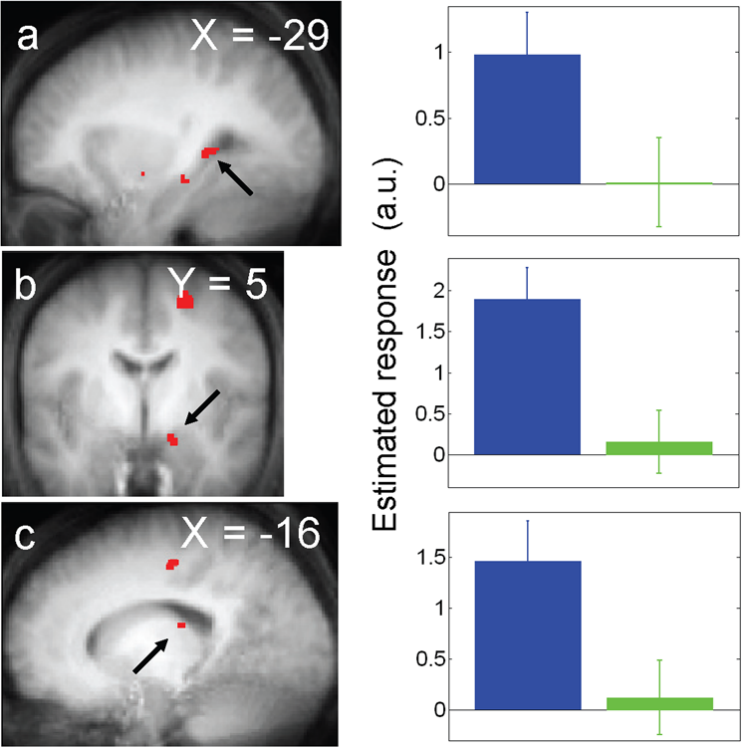
Significant differences between blue and green light conditions at light onset. *Left panels:* statistical results overlaid to the population mean structural image (p*_uncorrected_*<0.001). *Right panels.* Mean parameter estimates of the blue and green light conditions at light onset (arbitrary units±SEM). *a.* left hippocampus–*b.* right amygdala–*c.* left thalamus.

Two monochromatic light exposures were initiated 10 times in each session. This number of events was sufficient to conduct an analysis on the transient brain responses triggered by the onsets of the different light exposures. Significant differences between responses to blue and green light onsets were observed in two limbic areas, the left hippocampus and right amygdala, and in a location in the left thalamus, which was identical to that identified in the analysis of the sustained brain responses (Figure 3; Table 2). Activity estimates (Figure 3, right panels) show that these three brain areas strongly responded to blue light onsets while their activity was barely affected by green light onsets. No significant differences were found between violet and blue light onsets, while violet light onsets were found to increase left LGN activity significantly more than green light onsets (Table 2; Supplemental figure S2; Supplemental Tables S4).

**Table 2:** Light condition effects at light onset.

*Brain areas*	*xyz*	*Z*	*p*
***Blue light>green light***			
**Left hippocampus**	−28 −38 2	3.57	0.019
**Left thalamus**	−16 −24 18	3.37	0.034
**Right amygdala**	16 −4 −18	3.31	0.039
***Violet light>green light***			
**Left lateral geniculate nucleus**	−22 −22 −10	3.43	0.029

Coordinates (xyz) in the standard MNI space. No other significant light condition effects were found at light onset.

## Discussion

This study compared the brain responses elicited by violet, blue and green monochromatic light exposures of short duration (50 s) and equal irradiance (10^13^ ph/cm^2^/s) and presented in pairs of colors in 3 separate sessions. We hypothesized that these short light exposures would induce wavelength-dependent modulations of brain responses mainly in subcortical and brainstem areas. Accordingly, we report sustained wavelength-sensitive modulations of the regional brain responses evoked by a working memory task. In particular, blue light is superior to violet light in eliciting this response modulation. These results cannot be accounted for by any measurable difference in alertness or performance, nor by any order or expectation effect (Supplemental Data S1). These modulations are considered “sustained” because the brain activity is continuously enhanced during the 50 s blue light blocks and consistently so during the whole blue/violet fMRI session. Although sustained, these light-induced responses may be considered “early responses” when compared to the brain responses we reported after 18min of blue monochromatic light exposures [9]. As predicted, these early responses primarily involve subcortical areas related to arousal (brainstem and thalami). At the cortical level, the responses are enhanced in a single area, the MFG. This result contrasts with the enhanced responses in widespread cortical regions elicited by longer exposures [9] and suggest that the functional recruitment of the cortex requires longer exposures, and possibly the activating influence of subcortical structures.

An unexpected finding concerned the transient responses triggered at the onset of light exposures in two limbic areas, *i.e.* the amygdala and the hippocampus, and the left thalamus, irrespective of whether the subjects were engaged in the working memory task. These results are remarkable because blue light was superior to green light in eliciting these brain responses, even though illuminance was about 5 times higher for the green light.

Collectively, these sustained and transient responses show the efficacy of short wavelength (473nm) light in modulating brain activity, and indirectly suggest the involvement of melanopsin-expressing RGC, which are the photoreceptors most sensitive to this wavelength.

### Nonvisual versus visual responses

This study aimed at identifying brain and retinal mechanisms involved in early responses to light exposure that would likely be implicated in establishing the nonvisual responses that have been reported using longer duration exposures [2]–[11], [13], [14]. The experiment was not designed to ascertain whether or not these mechanisms constituted nonvisual responses to light. In fact we believe that the distinction between nonvisual and visual responses cannot be made in this experiment. Furthermore, there is growing evidence for a considerable overlap between visual and nonvisual photoreception systems. In rodents, rods and cones are involved in nonvisual responses to light [13], [27], [29], and non-expressing-melanopsin-expressing RGC project to nonvisual brain structures [24], [28]. On the other hand, melanopsin-expressing RGC project to structures typically involved in vision in rodents and primates [23]–[25], and appear to regulate visual processing both in rodents and in humans [53], [54]. Therefore, identifying a photoreceptor implicated in a response, does not directly inform on the nonvisual/visual nature of that response. In addition, in our protocol, participants' visual system was stimulated during light exposures, as participants obviously perceived the light. We minimized differences in photoreceptor stimulation by equating irradiance level across wavelengths. However, because stimulation of retinal photoreceptors changed with the wavelength of the exposure, signals transmitted to the visual system varied between wavelengths. Finally, we report effects of light at onset and after a few tens of seconds of exposure. From a human circadian biology perspective, this constitutes a very short time scale. During this period a mixed attenuation of cone signal and increase in intrinsic response of melanopsin-expressing RGC has been observed [25].

The sustained modulations of brain responses related to the task blocks arguably represent nonvisual responses. Indeed we report light-induced modulations of brain responses that are related to an auditory task and are most sensitive to blue light, which suggest the involvement of melanopsin-expressing RGC. In addition, whereas visual responses show quick attenuation [25], we detected modulations of brain responses that were maintained for 50 s. One could also argue that transient responses are nonvisual since they are likely to be predominantly mediated by melanopsin-expressing RGC. However, nonvisual responses are characterized by sustained activity modulation, and the transient effects we detected could therefore be considered to be mediated by the visual system. We believe that a more accurate description of our data is that we detected transient and sustained brain responses to light that appear to be predominantly mediated by melanopsin-expressing RGC, without qualification with respect to the visual or nonvisual nature of these responses.

### Sustained responses during task performance

A sustained enhancement of responses to the working memory task was observed during the exposures to blue, rather than violet light, in the brainstem, the thalamus, and the left MFG. No difference in response was observed when contrasting blue with green light, or violet with green light. These results suggest that the sustained response modulation by monochromatic light is most sensitive to blue light and least sensitive to violet light. The status of green light can not be precisely estimated but is consistent with an intermediate sensitivity. By inference, these results suggest that melanopsin-expressing RGC contribute most to these sustained responses to light. The observed, albeit smaller responses to violet and green light could be explained in two ways. According to the first interpretation they represent a combination of a weak contribution of S-cones and an intermediate involvement of M-cones. In line with this interpretation, melanopsin-expressing RGC and M-cones [13] seem to contribute greatly to nonvisual responses to light during the first minutes of the exposure in rodents. According to the second interpretation, melanopsin-expressing RGC are the only photoreceptors involved in the light induced modulations of brain activity and the observed smaller responses to violet and green light simply reflect the reduced sensitivity of melanopsin to these wavelengths. Melanopsin would then appear to have a greater sensitivity to wavelengths *longer* than 473 nm as compared to wavelengths *shorter* than 473 nm.

The brainstem area which was recruited by blue light corresponds tentatively to the LC. This result is important because it is the first time a brainstem structure is shown to respond to light in human. The LC may be a key structure in establishing effects of light. It could receive light information from the SCN, with which it is functionally connected in rodents [35]. As the major source of brain norepinephrine, it is in a position to modify the level of arousal [55], [56]. Finally, it is well established that the LC is involved in cognition and in executive functioning in particular [55].

Thalamic nuclei appear as the structures most consistently recruited in humans by “nonvisual” responses to light (polychromatic white light exposure [8]; monochromatic 470nm blue light exposure [9]). Like the brainstem, the thalamus is a key structure involved in the interaction between alertness and cognition in humans [57] and it is recruited by working memory tasks [58]. In addition, the thalamus might receive irradiance information through a two step pathway linking melanopsin-expressing RGC to the superior colliculus which in turn projects to the pulvinar [59].

Cortical responses were enhanced after recurring 50s periods of blue (relative to violet) monochromatic light exposure only in the left MFG, an area implicated in working memory [58]. This limited recruitment of cortical areas contrasts with our previous experiments, which used longer light exposures. Exposures to white light for about 21 minutes enhanced cortical responses to an auditory attention task in widespread cortical areas (dorso-lateral prefrontal cortex, IPS, superior parietal lobe, insula, precuneus, anterior and posterior cingulate cortices, and superior temporal gyrus [8]). Likewise, 18 min exposure to monochromatic blue (470 nm) light (as compared to green (550 nm) light) increased the responses induced by a working memory task in the left IPS, supramarginal gyrus, MFG, and right insula [9]. Collectively, these findings suggest that nonvisual responses require some time to build-up in the cortex. The assessment of this time course will require further studies characterizing the relations between photon density, duration of light exposure, and regional brain responses. Such studies will benefit from the methodological advances presented in this paper, namely within-session assessment of light-induced brain responses, which provide a fast, reliable technique to characterize light-induced brain responses.

Because there are no direct connections between nonvisual system and the cortex, we surmise that the light-induced enhancement of cortical responses follows indirect pathways involving activating subcortical structures.

### Transient responses to light onsets

An unanticipated result was the responses in left hippocampus, left thalamus, and right amygdala at light onsets of blue, relative to green light. Such differential response was not observed in the comparison between blue and violet lights or between violet and green lights. These results are surprising for several reasons. Because the visual system is most sensitive to green (555 nm) light [15], and since light onset is a typical visual stimulus, we expected green light to induce the greatest responses at onsets. In addition, M- and L-cones signals were reported to elicit ON responses in melanopsin-expressing RGC whereas S-cones were reported to mediate OFF responses [25]. Green light should therefore increase activity in these melanopsin-expressing RGC at light onset, and brain responses mediated by melanopsin-expressing RGC at light onset should be least sensitive to violet light.

Taken together, these elements suggest that melanopsin-expressing RGC contribute most to these transient limbic and thalamic responses to light onset. The reduced response sensitivity to violet and green lights could be explained in two ways. The contribution of M-cones could be considered as the weakest and the involvement of S-cones as intermediate, or melanopsin-expressing RGC could be deemed as the only photoreceptors involved, with a greater sensitivity to wavelengths *shorter* than 473nm as compared to *longer* than 473 nm wavelengths. Both assumptions could therefore suggest a shift in sensitivity between the transient brain responses related to light onsets and the sustained responses associated to task blocks. Accordingly, shift in wavelength sensitivity with exposure duration and intensity has been reported for circadian phase shift and pupillary constriction in rodents [13], [29]. However, in the brain areas showing responses most sensitive to blue light, changes in wavelength sensitivity are inferred based on comparisons with blue light, not on significant differences between violet and green lights. Characterizing duration/irradiance relationship will provide important data on changes in the involvement of the different retinal photoreceptors in eliciting brain responses to light.

Due to its anatomical connectivity, the amygdala is in good position to quickly receive irradiance information. The medial amygdala receives direct connections from melanopsin-expressing RGC in rodents [23]. In addition, a functional pathway linking the retina to the amygdala and bypassing the visual cortex through the superior colliculus and thalamus has been proposed in humans [59]. The hippocampus is connected to the amygdala [60], and both structures receive numerous afferents from the LC [61], a (potential) key component of nonvisual response system receiving indirect retinal projections [35].

At present, the functional significance of the limbic responses is unclear. However, it is tempting to suggest that blue light can modulate emotional processing by the amygdala. These effects may be related to the observed positive effects of long term light exposure regimes in seasonal affective disorder as well as in other psychiatric disorders [26]. Direct assessment of the influence of light on emotional processing should be used o further address this question.

Our protocol is very different from those used in vision neuroscience, because color vision investigations use isoluminant stimuli to account for luminance and brightness brain processing (*e.g.* Landisman and Ts'o, 2002; Tootell *et al.*, 2004). The significant difference in left LGN activity between violet and green light onset is therefore difficult to interpret. It is unlikely that it is related to the melanopsin-expressing RGC projections to the LGN found in Macaques [25], since it was not found in the session involving blue light.

### Conclusion

This study is part of a series of investigations of light processing in the entire human brain [7]–[9]. We demonstrate that a few tens of seconds of light induce immediate and significant wavelength-dependent changes in brain activity and that melanopsin-expressing RGC seem to provide the most important contribution to these changes. Our results also suggest specific pathways which relay light information from the retina to different brain areas and suggest that light can indirectly enhance cortical responses by recruiting structures in the brainstem and thalamus.

## Supporting Information

Data S1 (0.03 MB DOC)

Method S1 (0.02 MB DOC)

Table S1 (0.03 MB DOC)

Table S2 (0.04 MB DOC)

Table S3 (0.04 MB DOC)

Table S4 (0.04 MB DOC)

Figure S1 Behavioral results Mean values±SEM are plotted. The color of the light corresponds to the bar color. a. D-prime values in the different light conditions (2 sessions per condition) b. Criteria values in the different light conditions (2 sessions per condition) c. Reaction times in the different light conditions (2 sessions per condition) d. Sleepiness scores evolution across the protocol e. Sleepiness collected before each session type f. Sleepiness collected after each session type
(0.38 MB TIF)

Figure S2 Significant differences between green and violet light conditions at light onset in the left LGN. Left panels: statistical results overlaid to the population mean structural image (puncorrected<0.001). Right panels. Mean parameter estimates of the green and violet light conditions at light onset (arbitrary units±SEM) in the left LGN (−22 −22 −10).
(0.67 MB TIF)
