# Supplementary material for: Brain Responses to Violet, Blue, and Green Monochromatic Light Exposures in Humans: Prominent Role of Blue Light and the Brainstem
Source: PLoS One. 2007 Nov 28;2(11):e1247. doi: 10.1371/journal.pone.0001247 (PMC2082413; doi:10.1371/journal.pone.0001247)
Supplement: Data S1 — (0.03 MB DOC) [file pone.0001247.s001.doc]

## Behavior

## Accuracy

Subjects were instructed to be as accurate as possible and that at least 75% of correct responses were required. Mean accuracy was high for all sessions and light conditions (>93%) indicating that the task was easily performed throughout the protocol. Subjects could, however, sometimes execute the task differently and still reach high performance. We computed d-prime and criterion values to test this hypothesis. Sessions could not be directly compared because conditions changed from one session to the other. We therefore tested whether subjects’ behavior in one light condition changed from one session to the other and whether light conditions were similar.

Repeated measures ANOVA on d-prime values (Figure S1a) with light condition and session as within-subject factors revealed no main effects of light condition (F-value = 0.18; df = 2, 28; p-value = 0.84) and of session (F-value = 0.04; df = 1, 14; p-value = 0.84), and no light condition by session interaction (F-value = 0.18; df = 2,28; p-value =0.83). Repeated measures ANOVA on criterion values (Figure S1b) with light condition and session as within-subject factors revealed no main effects of light condition (F-value = 0.02; df = 2, 28; p-value = 0.98) and of session (F-value = 0.34; df = 1, 14; p-value = 0.57), and no light condition by session interaction (F -value = 0.24; df = 2, 28; p-value = 0.79).

## Reaction times

### Subjects were instructed to respond as fast as possible but to avoid anticipation errors, i.e. to prefer accuracy over speed. Mean reaction times for each light condition in each session were nevertheless analyzed for completeness of the results (Figure S1c). Again, sessions could not be directly compared because conditions changed from one session to the other. We therefore tested whether subjects’ reaction times in one light condition changed from one session to the other, and whether reaction times were similar across light conditions. Repeated measures ANOVA on mean reaction times with light condition and session as within-subject factors revealed no main effects of light condition (F-value = 0.72; df = 2, 28; p-value = 0.49) and of session (F-value = 0.28; df = 1, 14; p-value = 0.61), and no light condition by session interaction (F -value = 1.19 ; df = 2, 28; p-value = 0.32).

## Subjective sleepiness

Repeated measure ANOVA on KSS scores (Figure S1d) with repetition as within-subject factors revealed main effects of repetition (F-value = 10.22; df = 6, 84; p-value < 10^-6^). Planned comparisons showed significant differences in KSS scores collected before and after entering the scanner (F-value = 20.67; df = 1, 14; p-value = 0.0005) and between the KSS scores collected after the second session and those collected after the first and the third session (F-value = 6.67; df = 1, 14; p-value = 0.022). Apparently, entering the scanner and the change in posture and light level, was associated with significantly increased sleepiness. However, the randomization of session type order prevented this time effect on sleepiness from biasing our data. Indeed, repeated measure ANOVA with KSS scores collected before each session (Figure S1e) as within subject factor did not show significant differences (F-value = 0.28; df = 2, 28; p-value = 0.76), nor did the repeated measure ANOVA with KSS scores collected after each session (Figure S1f) as within subject factor (F-value = 0.10; df = 2, 28; p-value = 0.91).

## Experimental condition preferences

In order to rule out a placebo or expectation effect, we debriefed the participants about their color preference. Five subjects preferred the green illumination, six the blue light condition, four preferred the violet light. Therefore, differences in expectation or placebo effects are unlikely explanations for the results of this experiment.
