# Supplementary material for: Brain Responses to Violet, Blue, and Green Monochromatic Light Exposures in Humans: Prominent Role of Blue Light and the Brainstem
Source: PLoS One. 2007 Nov 28;2(11):e1247. doi: 10.1371/journal.pone.0001247 (PMC2082413; doi:10.1371/journal.pone.0001247)
Supplement: Method S1 — (0.02 MB DOC) [file pone.0001247.s002.doc]

## FMRI data

Because the melanopsin photoreception system is viewed as a “photon counter” integrating irradiance information over long periods of time, we hypothesized that some brain areas might see their activity build up during the 50s of illumination, irrespective of whether the task is performed or not, and then return to a baseline level once light is turned off. We therefore added “sawtooth-like functions” in the design matrix that modeled this build-up effect. However, these regressors did not reveal significant differences between light conditions (Supplemental tables S2). This supports the view that the differences between light conditions were stable during the 50s illuminations.
