# Supplementary material for: Brain Responses to Violet, Blue, and Green Monochromatic Light Exposures in Humans: Prominent Role of Blue Light and the Brainstem
Source: PLoS One. 2007 Nov 28;2(11):e1247. doi: 10.1371/journal.pone.0001247 (PMC2082413; doi:10.1371/journal.pone.0001247)
Supplement: Table S1 — (0.03 MB DOC) [file pone.0001247.s003.doc]

**Supplemental Tables S1. Light combination order across subjects**

**Bold:** Number of participants who were exposed to a given combination of wavelengths in their first, second, or third session.

*Italic:* number of participants exposed first to a particular wavelength in a given session.

|  | blue/green | violet/blue | violet/green |
| --- | --- | --- | --- |
| First session | **5**  *blue first: 3*  *green first: 2* | **5**  *violet first: 2*  *blue first: 3* | **5**  *violet first: 3*  *green first: 2* |
| Second session | **5**  *blue first: 2*  *green first: 3* | **6**  *violet first: 3*  *blue first: 3* | **4**  *violet first: 2*  *green first: 2* |
| Third session | **5**  *blue first: 4*  *green first: 1* | **4**  *violet first: 2*  *blue first: 2* | **6**  *violet first: 3*  *green first: 3* |
