# Supplementary material for: Brain Responses to Violet, Blue, and Green Monochromatic Light Exposures in Humans: Prominent Role of Blue Light and the Brainstem
Source: PLoS One. 2007 Nov 28;2(11):e1247. doi: 10.1371/journal.pone.0001247 (PMC2082413; doi:10.1371/journal.pone.0001247)
Supplement: Table S2 — (0.04 MB DOC) [file pone.0001247.s004.doc]

**Supplemental Tables S2**. **Light condition effects during the *2-back task***

These responses were not considered significant because they did not survive the correction for multiple comparisons either on the whole brain volume (no prior) or on a volume of interest centered on published coordinates (priors available).

***Blue light > Green light***

No significant voxel at p=0.001 *uncorrected.*

***Green light > Blue light***

No significant voxel at p=0.001 *uncorrected.*

***Violet light > Blue light***

| ***Brain areas*** | ***xyz*** | ***Z*** |
| --- | --- | --- |
| **Right hippocampus** | 28 -24 -12 | 3.33 |

***Blue light > Violet light***

| ***Brain areas*** | ***xyz*** | ***Z*** |
| --- | --- | --- |
| **Left precentral sulcus** | -38 -22 52 | 4.36 |
| **Right insula** | 36 -28 16 | 4.18 |
| **Left superior frontal gyrus** | -8 0 72 | 4.00 |
| **Right superior frontal sulcus** | 24 20 66 | 3.72 |
| **Right inferior frontal gyrus** | 56 22 4  38 48 40 | 3.68  3.52 |
| **Left insula** | -30 -28 14 | 3.53 |
| **Right middle occipital gyrus** | 32 -96 -8 | 3.49 |
| **Right superior precentral sulcus** | 44 12 34 | 3.32 |
| **Right superior temporal sulcus** | 48 -20 -16 | 3.32 |
| **Left lateral fissure** | -56 -22 14 | 3.18 |
| **Right lateral fissure** | 54 -24 10 | 3.16 |

***Green light > Violet light***

No significant voxel at p=0.001 *uncorrected.*

***Violet light > Green light***

| ***Brain areas*** | ***xyz*** | ***Z*** |
| --- | --- | --- |
| **Right middle occipital gyrus** | 56 -68 0 | 3.34 |
| **Left middle occipital gyrus** | -50 -82 9 | 3.69 |
