# Supplementary material for: Brain Responses to Violet, Blue, and Green Monochromatic Light Exposures in Humans: Prominent Role of Blue Light and the Brainstem
Source: PLoS One. 2007 Nov 28;2(11):e1247. doi: 10.1371/journal.pone.0001247 (PMC2082413; doi:10.1371/journal.pone.0001247)
Supplement: Table S3 — (0.04 MB DOC) [file pone.0001247.s005.doc]

**Supplemental Tables S3. Light condition effects modeled by saw-tooth-like regressors.**

These regressors model brain activity that show progressive build up during the whole 50s illumination. These responses were not considered significant because they did not survive the correction for multiple comparisons either on the whole brain volume (no prior) or on a volume of interest centered on published coordinates (priors available).

***Blue light >*** Green light

| ***Brain areas*** | ***xyz*** | ***Z*** |
| --- | --- | --- |
| **Right cuneus** | 16 -92 6 | 3.48 |

***Green light > Blue light***

No significant voxel at p=0.001 *uncorrected.*

***Violet light >*** Blue light

| ***Brain areas*** | ***xyz*** | ***Z*** |
| --- | --- | --- |
| **Left superior frontal gyrus** | -6 16 54 | 3.62 |
| **Right inferior frontal gyrus** | 42 24 20 | 3.40 |
| **Right lateral fissure** | 54 -22 8 | 3.41 |
| **Left insula** | -32 16 -8 | 3.37 |
| **Right parieto-occipital fissure** | 12 -66 36 | 3.30 |
| **Right superior temporal sulcus** | 42 -16 -14 | 3.29 |
| **Left lateral fissure** | -56 -24 14 | 3.25 |
| **Right cingulate sulcus** | 2 -46 46 | 3.18 |

***Blue light > Violet light***

No significant voxel at p=0.001 *uncorrected.*

***Green light >*** Violet light

| ***Brain areas*** | ***xyz*** | ***Z*** |
| --- | --- | --- |
| **Right central sulcus** | 64 -34 44 | 3.92 |
| **Right inferior frontal gyrus** | 52 40 4 | 3.34 |
| **Left middle occipital gyrus** | -52 -74 2 | 3.28 |

***Violet light >*** Green light

| ***Brain areas*** | ***xyz*** | ***Z*** |
| --- | --- | --- |
| **Left cuneus** | -8 -104 10 | 3.36 |
