# Supplementary material for: Brain Responses to Violet, Blue, and Green Monochromatic Light Exposures in Humans: Prominent Role of Blue Light and the Brainstem
Source: PLoS One. 2007 Nov 28;2(11):e1247. doi: 10.1371/journal.pone.0001247 (PMC2082413; doi:10.1371/journal.pone.0001247)
Supplement: Table S4 — (0.04 MB DOC) [file pone.0001247.s006.doc]

**Supplemental Tables S4. Light condition effects at light onsets.**

These responses were not considered significant because they did not survive the correction for multiple comparisons either on the whole brain volume (no prior) or on a volume of interest centered on published coordinates (priors available).

***Blue light > Green light***

| ***Brain areas*** | ***xyz*** | ***Z*** |
| --- | --- | --- |
| **Right superior frontal sulcus** | 24 -4 60  18 2 64 | 4.34  4.11 |
| **Left insula** | -38 -10 16 | 4.19 |
| **Left middle occipital gyrus** | -20 -88 6 | 3.54 |
| **Left hippocampus** | -28 -24 -14 | 3.35 |
| **Left superior frontal gyrus** | -6 -10 60 | 3.22 |

***Green light > Blue light***

No significant voxel at p=0.001 *uncorrected.*

***Violet light >*** Blue light

| ***Brain areas*** | ***xyz*** | ***Z*** |
| --- | --- | --- |
| **Left cerebellum** | -18 -82 -26 | 3.81 |
| **Right superior temporal sulcus** | 66 -34 -10 | 3.66 |
| **Left inferior frontal sulcus** | -28 42 14 | 3.64 |
| **Left superior frontal sulcus** | -28 -8 52 | 3.59 |
| **Left hippocampus** | -34 -10 -32  -24 -28 -10 | 3.58  3.17 |
| **Right posterior cingulate cortex** | 2 -42 44 | 3.50 |
| **Right angular gyrus** | 42 -64 30 | 3.39 |
| **Right parieto-occipital fissure** | 26 -60 6 | 3.37 |

***Blue light > Violet light***

No significant voxel at p=0.001 *uncorrected.*

***Violet light > Green light***

| ***Brain areas*** | ***xyz*** | ***Z*** |
| --- | --- | --- |
| **Right hippocampus** | 28 -38 4 | 3.30 |

***Green light > Violet light***

| ***Brain areas*** | ***xyz*** | ***Z*** |
| --- | --- | --- |
| **Right parahippocampus** | 26 -40 -14 | 3.92 |
| **Left precuneus** | -2 -42 58 | 3.82 |
| **Left anterior cingulate cortex** | -6 52 4 | 3.81 |
| **Right inferior frontal gyrus** | 62 22 2 | 3.38 |
| **Left posterior cingulate cortex** | -6 -24 42 | 3.18 |
