# Supplementary figures and images for: Brain Responses to Violet, Blue, and Green Monochromatic Light Exposures in Humans: Prominent Role of Blue Light and the Brainstem
Source: PLoS One. 2007 Nov 28;2(11):e1247. doi: 10.1371/journal.pone.0001247 (PMC2082413; doi:10.1371/journal.pone.0001247)

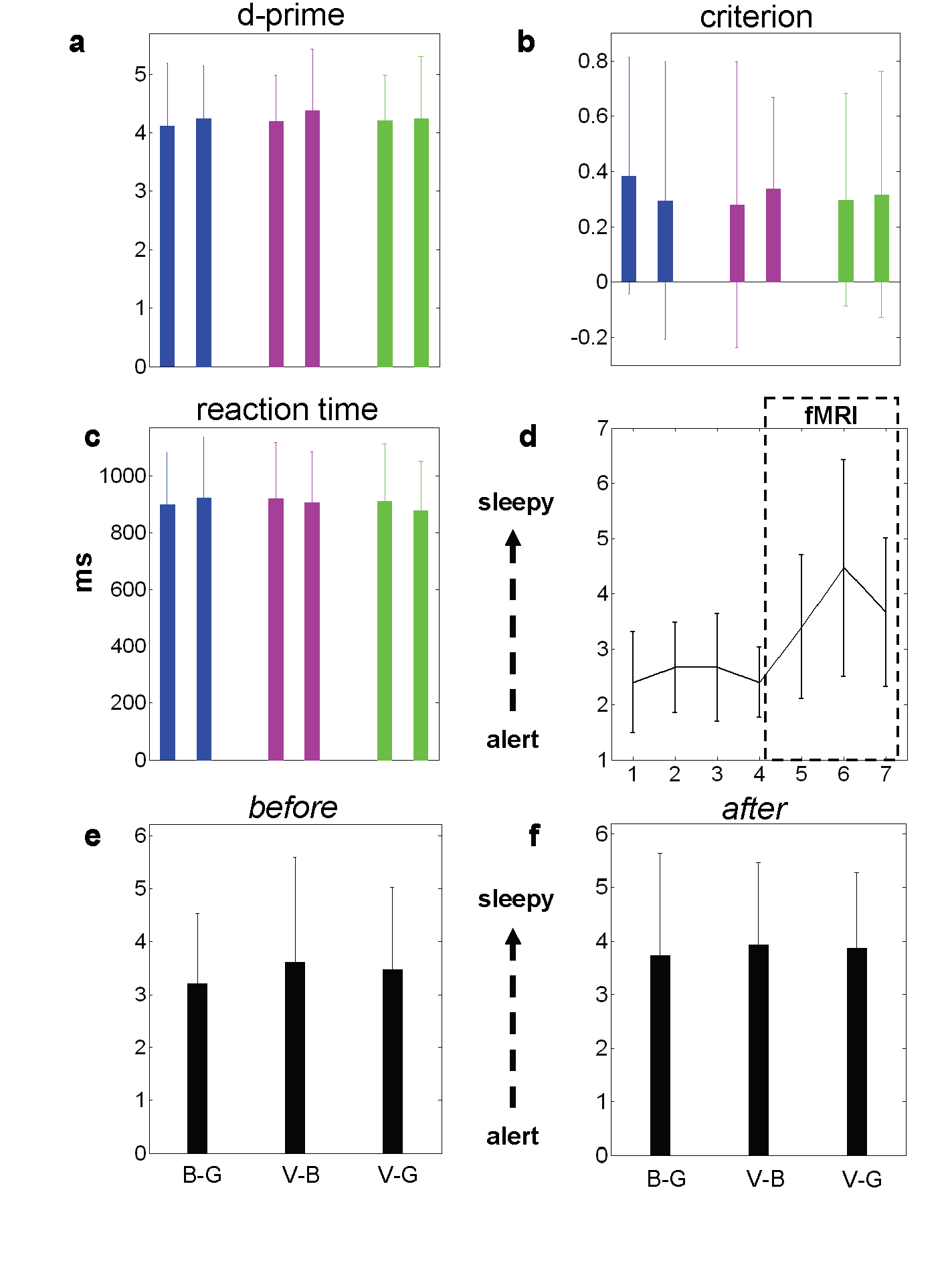

Supplement: Figure S1 — Behavioral results Mean values±SEM are plotted. The color of the light corresponds to the bar color. a. D-prime values in the different light conditions (2 sessions per condition) b. Criteria values in the different light conditions (2 sessions per condition) c. Reaction times in the different light conditions (2 sessions per condition) d. Sleepiness scores evolution across the protocol e. Sleepiness collected before each session type f. Sleepiness collected after each session type (0.38 MB TIF) [file pone.0001247.s007.tif]

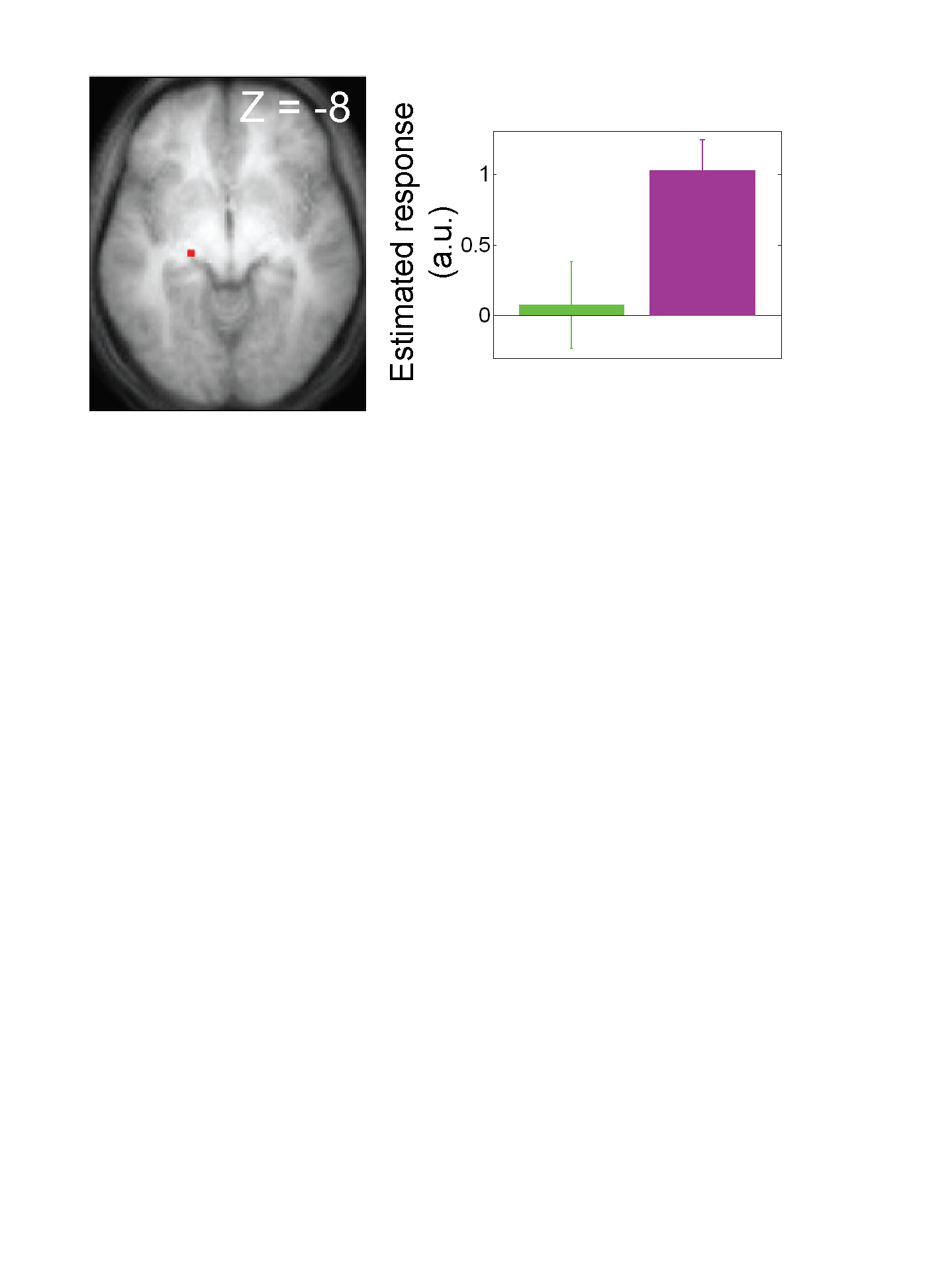

Supplement: Figure S2 — Significant differences between green and violet light conditions at light onset in the left LGN. Left panels: statistical results overlaid to the population mean structural image (puncorrected<0.001). Right panels. Mean parameter estimates of the green and violet light conditions at light onset (arbitrary units±SEM) in the left LGN (−22 −22 −10). (0.67 MB TIF) [file pone.0001247.s008.tif]
